# HEALTH DISPARITIES: Climate Change and Health: A Native American Perspective

**DOI:** 10.1289/ehp.118-a64

**Published:** 2010-02

**Authors:** Bob Weinhold

**Affiliations:** **Bob Weinhold**, MA, has covered environmental health issues for numerous outlets since 1996. He is a member of the Society of Environmental Journalists

The intricate, intertwined forces driving global climate change are mirrored by similar complexity in the human response to it. That makes it nearly impossible to anticipate the stance of any one group based solely on a label such as nationality, race, or economic class. But there is ample evidence that the raw drive for survival—the ultimate environmental health perspective—is a common thread that often compels people to change their behavior. That is the case today for some Native Americans who are feeling the effects of dislocation and food shortages they attribute to climate change.

Native American populations comprise 564 federally recognized tribes and 70 additional tribal entities recognized by 16 states. They make up about 1% of the U.S. population and occupy about 4% of the land. There apparently have been no national climate change polls of this diverse group. But feedback from selected Native American individuals, organizations, and tribes indicates they hold the same full spectrum of opinions that exists within the rest of the country.

Among indigenous peoples in North America, the Native Americans who continue to practice traditional and subsistence lifestyles to perhaps the highest degree are those in Alaska, where 80% of the diet comes from the immediate surroundings, says Jose Aguto, policy advisor on climate change, environment, and natural resources for the National Congress of American Indians. Between 2002 and 2007, coastal erosion more than doubled along a 40-mile stretch of Alaska’s Beaufort Sea, according to a U.S. Geological Survey study in the 14 February 2009 issue of *Geophysical Research Letters*. The authors posited that numerous climate-related factors may be working together to change erosion patterns. Some coastal villages have been swamped, and substantial shifts are occurring in plant and animal populations as they try to adapt to the thawing tundra.

Peoples in Alaska have been some of the most vocal about climate change, says Del Laverdure, a member of the Crow tribe in Montana and deputy assistant secretary with the Bureau of Indian Affairs. Other tribes that have been active on the climate change issue include some in the Pacific Northwest, such as the Quinault Indian Nation in Washington, in response to changes in fish and forests that have already appeared, he adds.

The mindset of Native Americans who prefer a lifestyle that reflects traditional, lower-impact ways of living on the land and who take a dim view of those who pursue energy-intensive, consumer-oriented lifestyles is captured in the Mystic Lake Declaration, released 21 November 2009 following a Minnesota workshop sponsored by the National Aeronautics and Space Administration in conjunction with several Native American groups including Honor the Earth, a grassroots advocacy group. Among the many recommendations in the document, education of tribal members and their communities “is the number one thing,” says Carrie Billy, a Navajo from Arizona and president of the American Indian Higher Education Consortium.

That consortium supports 36 tribal colleges and universities serving nearly 30,000 full- and part-time students. This spring about one-third of these schools, funded in part by the National Science Foundation, will begin offering an introductory course comparing indigenous knowledge with western science understanding of climate processes and effects, and exploring potential mitigation and adaptation strategies for Native American Nations. Many of the other schools are likely to soon add this course, and some schools are already incorporating climate change information into other courses and projects. Billy says educating thousands of students beginning as early as kindergarten—who likely will in turn educate their communities—is essential if tribes are to effectively address climate change.

For Winona LaDuke, executive director of Honor the Earth and a member of the Ojibwe tribe in Minnesota, one of the highest priorities is to continue battling the fossil fuel and nuclear power industries, some of which are all too familiar to her. “Tribal governments are some of the biggest carbon dioxide producers in the world,” she says. Other high priorities include restoring indigenous seed varieties that are more tolerant of drought and other climate extremes, and developing wind power, a technology already advancing rapidly throughout the Great Plains, according to the National Renewable Energy Laboratory.

For some Native Americans, traditional knowledge developed over millennia of living on specific lands has been rendered almost meaningless, with many tribes evicted from the ecosystems they historically occupied and confined to reservations, sometimes on harsh, unproductive land. Forced to cope with rugged conditions, some tribes have discovered in recent decades that beneath their lands lie valuable resources of great economic value: coal, oil, and natural gas. The fact that these are major emitters of carbon dioxide and other pollutants when burned is outweighed in the minds of some members of these tribes by the immediate economic rewards.

“Environmental issues are at the bottom,” says Pete Homer, a member of the Mojave tribe in the Southwest and president of the National Indian Business Association. “We’ve got too many other problems, like poverty and a lack of jobs. We got to create that economic base on the reservations. We hear about climate change. But our members tell us it’s not much of a problem. They haven’t seen anything that is going to hurt them.” In fact, on 29 September 2009 *The Arizona Republic* reported the Hopi Tribal Council had adopted a resolution condemning several environmental advocacy groups for pushing for closure of the coal-fired Navajo Generating Station. The paper quoted tribal counsel Scott Canty as saying the plant and the coal mines that fuel it provide more than 70% of the Indian nation’s revenue.

Congress and federal agencies are in the early stages of discussions on how to deal with climate change, and Native Americans have been acknowledged as needing some unique attention in those efforts. One reason is that tribes are locked into their reservation land, limiting their adaptation options, says Christine Glunz, a spokewoman for the White House Council on Environmental Quality (CEQ). At the same time, Laverdure says it will be useful to include Native Americans in legislative and agency discussions so the tribes can share their hard-won ecological and resource management knowledge accumulated over the centuries. Legislation is beginning to work its way through Congress, and the CEQ is co-chairing an effort involving more than 20 federal agencies, departments, and offices to discuss a coordinated approach for mitigation and adaptation, with a progress report expected in the fall of 2010.

Some federal agencies appear to be willing to at least accept Native American input. “[R]ight now we are just listening to the tribes on climate change issues,” says U.S. Environmental Protection Agency spokeswoman Cathy Milbourn. “We don’t have a lot to say since this is in the very beginning stages of our discussions.”

As talks continue, many Native Americans are insisting their rights as sovereign nations be recognized. The status of federally recognized tribes as sovereign nations has long been on the books but has also long been a point of contention. However, that status will allow Native Americans to have the same access as states to financial and technological resources, and to have the same standing when dealing with federal regulations, says Jerry Pardilla, a member of the Penobscot Nation in Maine and executive director of the National Tribal Environmental Council, formed in 1991 and now supported by about 190 tribes.

Those are concepts Laverdure says he supports, and which are included in some way in the developing legislation. But how any financial aid to Native Americans is paid for is uncertain, he acknowledges, since there still are intense discussions over whether those who contribute the most to climate change problems should pay, or if victims of climate change must fend for themselves. “It’s a domestic example of what [heads of state] are dealing with in Copenhagen right now,” he said during the second week of the international climate change conference in that city, referring to the battles between poorer countries that often have contributed the least to global warming but are sometimes feeling the most effects, and richer countries that frequently are the highest emitters. He says, though, that Native Americans with coal, oil, or natural gas resources likely will have a bit of leverage in internal U.S. negotiations because as sovereign nations they have a role in determining how, or whether, they develop and burn those fuels.

But simply negotiating good deals with Congress and federal agencies isn’t the only challenge for Native Americans. “Many tribes do not have the organizational infrastructure and capacity to address the impacts of climate change upon their natural resources and physical infrastructure,” Aguto says. “Although thirty-two states have adopted or are in the process of developing climate change action plans, only one tribe has formally done so, although additional tribes have taken some related measures. Some of these circumstances can be attributed to historical neglect and a lack of funding from the federal government. The tribes are working to change this template through the proposed climate legislation.”

It will take several years to see how organizations such as tribes, federal agencies, and Congress decide how to address climate change. Meanwhile, those who are feeling the heat already likely will continue to follow their basic instincts. “I don’t have anything better to do than to try to survive,” LaDuke says.

## Figures and Tables

**Figure:**
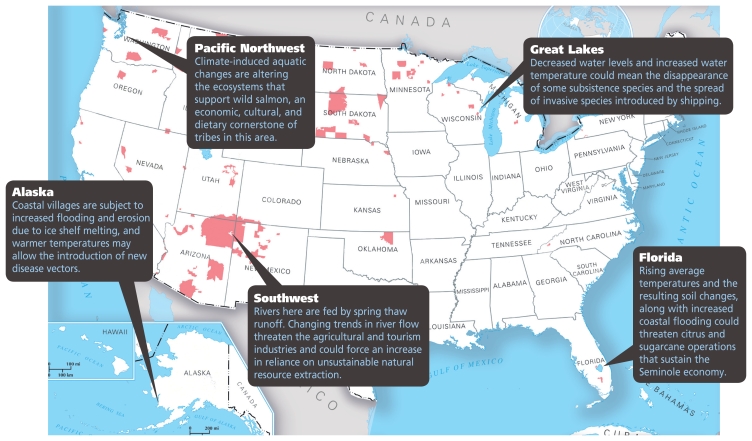
Climate Change Affects Tribes Nationwide **Tribal lands are indicated in pink.** Sources: map—www.nationalatlas.gov; climate change effect predictions—Hanna JM. 2007. Native communities and climate change: protecting tribal resources as part of national climate policy. Boulder, CO: Natural Resources Law Center / NWF. 2007. Overview of recent research: effects of global warming on the Great Lakes [fact sheet]. Ann Arbor, MI: National Wildlife Federation.

